# Virion Structure of Iflavirus Slow Bee Paralysis Virus at 2.6-Angstrom Resolution

**DOI:** 10.1128/JVI.00680-16

**Published:** 2016-07-27

**Authors:** Sergei Kalynych, Antonín Přidal, Lenka Pálková, Yevgen Levdansky, Joachim R. de Miranda, Pavel Plevka

**Affiliations:** a Structural Virology, Central European Institute of Technology, Masaryk University, Brno, Czech Republic; b Department of Zoology, Fishery, Hydrobiology, and Apidology, Faculty of Agronomy, Mendel University in Brno, Brno, Czech Republic; c Department of Ecology, Swedish University of Agricultural Sciences, Uppsala, Uppsala, Sweden; Hudson Institute of Medical Research

## Abstract

The western honeybee (Apis mellifera) is the most important commercial insect pollinator. However, bees are under pressure from habitat loss, environmental stress, and pathogens, including viruses that can cause lethal epidemics. Slow bee paralysis virus (SBPV) belongs to the Iflaviridae family of nonenveloped single-stranded RNA viruses. Here we present the structure of the SBPV virion determined from two crystal forms to resolutions of 3.4 Å and 2.6 Å. The overall structure of the virion resembles that of picornaviruses, with the three major capsid proteins VP1 to 3 organized into a pseudo-T3 icosahedral capsid. However, the SBPV capsid protein VP3 contains a C-terminal globular domain that has not been observed in other viruses from the order Picornavirales. The protruding (P) domains form “crowns” on the virion surface around each 5-fold axis in one of the crystal forms. However, the P domains are shifted 36 Å toward the 3-fold axis in the other crystal form. Furthermore, the P domain contains the Ser-His-Asp triad within a surface patch of eight conserved residues that constitutes a putative catalytic or receptor-binding site. The movements of the domain might be required for efficient substrate cleavage or receptor binding during virus cell entry. In addition, capsid protein VP2 contains an RGD sequence that is exposed on the virion surface, indicating that integrins might be cellular receptors of SBPV.

**IMPORTANCE** Pollination by honeybees is needed to sustain agricultural productivity as well as the biodiversity of wild flora. However, honeybee populations in Europe and North America have been declining since the 1950s. Honeybee viruses from the Iflaviridae family are among the major causes of honeybee colony mortality. We determined the virion structure of an Iflavirus, slow bee paralysis virus (SBPV). SBPV exhibits unique structural features not observed in other picorna-like viruses. The SBPV capsid protein VP3 has a large C-terminal domain, five of which form highly prominent protruding “crowns” on the virion surface. However, the domains can change their positions depending on the conditions of the environment. The domain includes a putative catalytic or receptor binding site that might be important for SBPV cell entry.

## INTRODUCTION

The western honeybee Apis mellifera plays a vital role in agriculture by providing pollination services for numerous food crops, especially those with high nutritional and economic value (1). Honeybees are also critical for maintaining the ecological and genetic diversity of wild flowering plants (2). In addition, bumblebees and several other solitary bee species are becoming increasingly important commercial pollinators of specific crops (3). However, bees and the pollination services they provide are under increasing stress due to habitat loss, intensified agricultural management, pesticides, parasites, and pathogens, including numerous viruses (3). Annual honeybee colony mortality has been increasing in North America and Europe over the last couple of decades (4), which, coupled with a long-term decline in beekeeping, has become a serious threat to the adequate provision of pollination services and food security (4–6).

Honeybees are hosts to a large number of viruses, most of which persist covertly within the honeybee population interrupted by occasional outbreaks. Such outbreaks of some of the viruses can have fatal consequences for individual workers and whole colonies (7). Colony collapse disorder (CCD), a still largely unexplained rapid loss of adult bees from colonies, has been linked to virus infections (8, 9). Much of winter honeybee colony mortality is also associated with viruses (10, 11). The viruses that have the greatest impact on honeybee populations are small icosahedral picorna-like viruses from the families Dicistroviridae and Iflaviridae, including slow bee paralysis virus (SBPV), sacbrood virus (SBV), deformed wing virus (DWV), and varroa destructor virus 1 (VDV-1) (7). SBPV was discovered in 1974 (12) and was linked to honeybee colony mortality in the United Kingdom in the 1980s (13). Despite its efficient transmission by Varroa destructor (14), SBP is a rare disease of honeybees (15). However, it is common in bumblebees (16, 17), and therefore, honeybees may be an incidental, secondary host.

Viruses from the order Picornavirales have nonenveloped icosahedral virions containing a single-stranded positive-sense RNA genome about 10,000 nucleotides long (18). Picornavirus genomes are translated into polyproteins that are co- and posttranslationally cleaved by viral proteases to produce structural (capsid-forming) and nonstructural proteins. The capsid proteins originating from a single polyprotein form a protomer—the basic building block of the capsid. The entire capsid consists of 60 such protomers, arranged in 12 pentamer units of five protomers each. The major capsid proteins VP1 to VP3 are arranged in a pseudo-T3 icosahedral capsid.

The only structural information available on Iflaviridae family members is the 25-Å resolution cryo-electron microscopy structure of the Chinese sacbrood virus (19). The structure confirmed the pseudo-T3 icosahedral symmetry of the capsid and revealed a smooth outer surface of the virion. Iflaviruses were proposed to harbor short VP4 subunits consisting of only about 20 residues (15, 20). However, because of the low molecular weight of the peptides, the existence of VP4 subunits has not been unequivocally established (15, 20). Previous genetic and proteomic analyses of iflaviruses revealed a C-terminal extension of about 160 residues in length of one of the capsid proteins (15, 20, 21). Here we present the structure of SBPV determined from two crystal forms to resolutions of 3.4 Å and 2.6 Å. The structures offer the first high-resolution snapshots of a virus from the family Iflaviridae and of a viral pathogen of the honeybee.

## MATERIALS AND METHODS

### Virus propagation in honeybee pupae.

Propagations of SBPV were carried out as described in the COLOSS BEEBOOK (22). Brood areas with Apis mellifera white-eyed pupae were identified by color and structural features of the cell caps. White-eyed pupae were carefully extracted from the brood combs, so as not to injure the pupae. The pupae were placed on paper furrows with their ventral side up. In total, 544 pupae were used for the SBPV propagation. A virus inoculum (1 μl) was injected into pupae with a Hamilton micropipette with a 30-gauge 22-mm-long needle through the intersegmental cuticle between the 4th and 5th sternites. Pupae that leaked hemolymph after the injection were discarded. The optimal concentration of the virus in the inoculum for virus production was determined experimentally by comparing virus yields when using different virus concentrations in the injection inoculum. Inoculated pupae were placed into petri dishes with the paper furrows and incubated at 30°C and 75% humidity for 5 days. After incubation, the pupae were frozen at −20°C. For long-term storage, the pupae were kept at −80°C.

### Virus purification.

Fifty to 70 experimentally infected honeybee pupae were homogenized with a Dounce homogenizer in 30 ml of phosphate-buffered saline (PBS), pH 7.5 (Sigma-Aldrich). The nonionic detergent NP-40 was added to a final concentration of 0.5%, and the homogenate was incubated for 1 h at room temperature. The extract was centrifuged at 8,000 × *g* for 30 min. The pellet was discarded and the supernatant was centrifuged at 150,000 × *g* for 3 h in a Ti50.2 fixed-angle rotor (Beckman-Coulter). The resulting pellet was resuspended in PBS to a final volume of 5 ml. MgCl_2_ was added to a final concentration of 5 mM, along with 20 μg/ml of DNase I and 20 μg/ml of RNase. The solution was incubated at room temperature for 30 min and centrifuged at 4,000 × *g* for 15 min. The resulting supernatant was loaded onto a CsCl (0.6-g/ml) solution prepared in PBS. The ultracentrifugation proceeded for 16 h to establish the CsCl gradient. Virus bands were collected by gentle piercing of the ultracentrifuge tubes with an 18-gauge needle. The viruses were transferred to PBS by several rounds of concentration and dilution using centrifuge filter units with a 100-kDa-molecular-mass cutoff. This procedure yielded about 300 μg of virus with a purity sufficient for sparse-matrix crystallization screening experiments. Sample purity with respect to contaminating honeybee viruses was checked by reverse transcription-quantitative PCR (RT-qPCR) using previously reported virus-specific assays (22). In both preparations, the total sum of contaminating viruses was less than 1% of the virus of interest. The nucleotide sequences of the virus preparations were determined by sequencing 300 ng of RNA, purified using a Qiagen RNA purification kit, by IonTorrent technology and standard protocols for library preparation and sequencing. The IonTorrent reads were mapped to the SBPV GenBank reference sequence EU035616 (SBPV) using Tmap v4.4.8, included in TorrentSuite 4.4.2, with Life Technologies-recommended parameters. Variability and consensus sequences were created using mpileup from samtools v.0.1.8 and an in-house script.

### SBPV crystallization.

SBPV crystallization screening was conducted at 4°C and 20°C with virus concentrations of 5 mg/ml and 10 mg/ml. In total, 2,100 conditions were tested in a 96-well, sitting-drop vapor diffusion format. The initial crystals that formed in 0.1 M sodium citrate (pH 6.5)–5% (wt/vol) polyethylene glycol 4000 (PEG 4000) after 7 days of incubation at 20°C were spherical, with diameters of less than 0.03 μm. The crystallization conditions were optimized by using a 96-well additive screen (Hampton Research Inc.). Optimized crystals with cubic morphology grew under the starting conditions with extra 0.2 M NDSB-221 (nondetergent sulfobetaine) and could be reproduced in a hanging-drop format by mixing 1.5 μl of 10-mg/ml purified virus solution with 0.5 μl of the reservoir solution. The optimized crystals were cubic and required 3 weeks to reach their final size of about 0.1 μm. The best diffraction was obtained when crystals were transferred to a reservoir solution containing 10% ethylene glycol prior to flash-freezing in liquid nitrogen. Out of approximately 200 crystals tested, two crystals diffracted X rays to a resolution of 3.4 Å.

Another crystal form was discovered at 4°C in 0.1 M sodium acetate (pH 4.5)–5% PEG 10000 and contained rectangular crystals of about 0.1 mm. The crystals could be reproduced in a hanging-drop format, with some crystals reaching a length of 0.3 mm. The crystals were subjected to dehydration by gradually transferring the coverslip containing the hanging drop to the reservoir solution containing increasing concentrations of sodium acetate (pH 4.5) and of PEG 10000 as described previously (23). At 20% PEG 10000, crystals were harvested, cryoprotected in mother liquor solution containing 20% glycerol, and flash-frozen in liquid nitrogen. Out of 50 crystals screened, two crystals diffracted X rays to a resolution of 2.6 Å.

### SBPV structure determination and refinement.

Diffraction data from SBPV crystal form 1 were collected at the Swiss Light Source X06SA beamline equipped with Pilatus-6M detector at the wavelength of 1.00003 Å at 100 K using a 0.1° rotation per image. The crystals were of space group I23. Unit cell size and packing considerations indicated that one pentamer of capsid protein protomers occupied a crystallographic asymmetric unit. There are two possibilities for superimposing icosahedral 532 symmetry with the 23 symmetry of the crystal, which are perpendicular to each other. The orientation of the virion was determined from a plot of the 5-fold rotation function, calculated with the program GLRF (24). Reflections between 5.0-Å and 4.5-Å resolutions were used for the calculations. Because of the superposition of the icosahedral and crystallographic symmetry, the center of the particle had to be positioned at the intersection of the 2-fold and 3-fold symmetry axes of the crystal. The triatoma virus (TrV) structure (PDB code 3NAP), converted to polyalanine, was used as a molecular replacement model. The model was placed into the orientation and position in the unit cell as described above and used to calculate phases for reflections at up to a 10-Å resolution, using the program CNS (25). The model-derived phases were refined by 25 cycles of 5-fold real-space electron density map averaging using the program ave (26). The mask for electron density averaging was generated by including all voxels within 5 Å of any atom of the TrV model, using the program mama from the package USF (27). Phase extension was applied in order to obtain phases for higher-resolution reflections. The addition of a small fraction of higher-resolution data (one index at a time) was followed by three cycles of averaging. This procedure was repeated until phases were obtained for all the reflections, up to a resolution of 3.4 Å. Inspection of the map showed that the mask used for electron density averaging cut the electron density of the capsid in an area around the icosahedral 5-fold axis. Thus, a new mask was prepared based on a correlation map calculated by comparing the electron density distributions among the five noncrystallographic symmetry (NCS)-related icosahedral asymmetric units. The correlation map was calculated using the program coma from Uppsala Software Factory (USF) (28). A cutoff value of 0.5 was used for the inclusion of voxels into the mask. The surface of the correlation mask was smoothened using the program mama (28). The phase extension procedure was repeated using the new mask. The resulting map was of sufficient quality to allow model building.

The program Buccaneer was used for automated model building, utilizing the 5-fold NCS present in the crystal (29, 30). The model from the automated building was about 50% complete, with assigned amino acid sequences. The initial model was subjected to iterative manual rebuilding using the programs Coot and O (31, 32) and coordinate and B-factor refinement using the programs CNS (25) and Phenix (33). No water molecules were added due to the limited resolution of the diffraction data.

Diffraction data from SBPV crystal form 2 crystals were collected at the synchrotron Soleil Proxima-1 beamline equipped with the Pilatus-6M detector at a wavelength of 0.97857 Å at 100 K using a 0.1° rotation per image. The crystals were of space group I222. The unit cell dimensions and the virus packaging considerations indicated that the crystallographic asymmetric unit consists of three pentamers of capsid protein protomers. Initially, a pentamer corresponding to the entire atomic model of crystal form I was used as a molecular replacement model to find the orientation and translation of the three pentamers in the crystallographic asymmetric unit using the program Phaser (34). The initial electron density map was subjected to 30 cycles of noncrystallographic symmetry averaging using the program AVE (26) and employing the mask based on the model from crystal form I. The averaged map lacked the electron density corresponding to the protruding domain altogether, which suggested that the molecular mask did not cover the correct part of the map. Therefore, a correlation map was calculated, as described for crystal form I, and the mask based on the correlation map was used for averaging. This map was used for the automated model building in the program Buccaneer (29) from the CCP4i software suite (35). The geometry of the model was adjusted manually using the program Coot (32). The coordinate and B-factor refinement were carried out using the program CNS (25) employing strict NCS constraints.

In order to improve the structure of the P domain in crystal form I, the P domain determined from crystal form II was positioned in crystal I using the program Phaser (34). The model of the icosahedral asymmetric unit with the properly positioned P domain was then used to generate a new mask for real-space electron density averaging in the program mama (27). Thirty cycles of real-space electron density averaging were carried out using the program AVE (26). P domain residues with no corresponding density in the averaged map were manually removed using the program Coot (32). The model was subjected to coordinate and B-factor DEN-assisted refinement using the atomic model of crystal form 2 as a reference structure using the software package CNS (25, 36).

### Protein structure accession numbers.

The atomic coordinates of the SBPV virion in crystal forms 1 and 2, together with the structure factors and phases obtained by phase extension, were deposited into the Protein Data Bank under codes 5J96 and 5J98, respectively.

## RESULTS AND DISCUSSION

### Structure of SBPV virion and capsid proteins.

**FIG 1:**
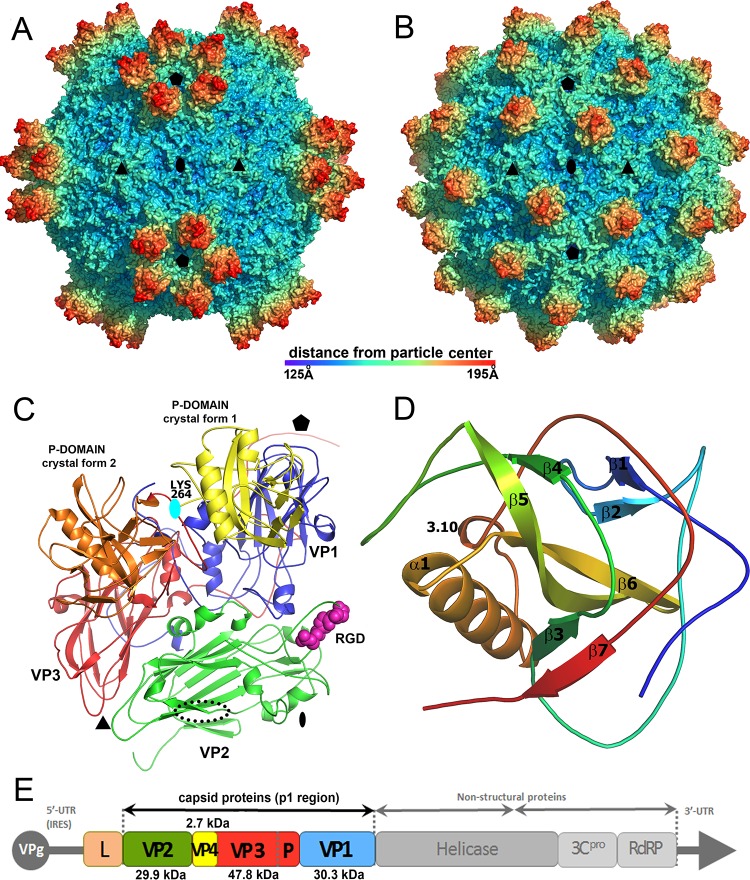
Structure of SBPV virion and icosahedral asymmetric unit. Surface representations of SBPV virions determined in crystal form 1 (A) and crystal form 2 (B) show differences in the positioning of the P domains. The surfaces of the particles are rainbow-colored based on the distance from the particle center. Depressions are shown in blue and peaks in red. (C) Cartoon representation of SBPV icosahedral asymmetric unit. VP1 is shown in blue, VP2 in green, and VP3 in red. The P domain positioned as in crystal form 1 is shown in yellow and in crystal form 2 in orange. Locations of 5-fold, 3-fold, and 2-fold icosahedral symmetry axes are indicated by a pentagon, triangle, and oval, respectively. The RGD motif found in the GH loop of VP2 subunit is shown as space-filling model in magenta. The position of the RGD motif in FMDV is indicated with a dotted black oval. The cyan oval indicates the position of rotation axis relating the two P domain positions. (D) Cartoon representation of P domain rainbow colored from the N terminus in blue to the C terminus in red. Names of secondary structure elements are indicated. (E) Diagram of SBPV genome organization. Capsid proteins VP1, VP2, and VP3 were identified based on their location in the capsid according to the picornavirus convention. Predicted molecular masses of capsid proteins are specified. The location of the P domain of VP3 is indicated. VPg, viral protein, genome linked; L, leader peptide; IRES, internal ribosome entry site; UTR, untranslated region; 3C^PRO^, 3C protease; RdRP, RNA-dependent RNA polymerase.

The structure of SBPV was determined from two crystal forms to resolutions of 3.4 Å and 2.6 Å (Table 1). The two structures are similar, with a Cα-atom root mean square deviation (RMSD) of 0.27 Å; however, they differ in the positions of protruding (P) domains of the VP3 subunits on the virion surface (Fig. 1A and B). The maximum outer diameter of the virion is 388 Å. The virion is bigger than those of other picornaviruses because of the P domains. The organization of capsid proteins within the SBPV virion is similar to that of other viruses from the order Picornavirales (Fig. 1C). The capsid is built from major capsid proteins VP1 to 3 arranged in pseudo-T3 icosahedral symmetry (Fig. 1). The major capsid proteins have jellyroll β-sandwich folds with β-strands named according to the picornavirus convention B to I (37). The two antiparallel β-sheets forming the β-sandwich fold contain the strands BIDG and CHEF, respectively. The structures of the major capsid proteins could be built except for residues 253 to 266 of VP1, 92 to 100 and 261 of VP2, and 418 to 430 of VP3. The electron density corresponding to VP4 could not be identified in either of the two structures.

**TABLE 1:** Crystallographic data collection and refinement statistics

Parameter	Crystal form 1	Crystal form 2
Crystallization conditions	Sodium citrate (pH 6.5), 5% (vol/vol) PEG 4000, 0.2 M NDSB-221	Sodium acetate (pH 4.5), 5% (vol/vol) PEG 10000
Space group	I23	I222
a, b, c (Å)	360.7, 360.7, 360.7	340.0, 396.8, 431.7
α, β, γ (**°**)	90, 90, 90	90, 90, 90
Resolution (Å)^*a*^	70.7–3.41 (3.45–3.41)	49.5–2.6 (2.64–2.60)
*R*_merge_^*a*^	0.31 (1.26)	0.20 (0.98)
<I>/<σI>^*a*^	5.6 (0.4)	6.0 (0.9)
Completeness (%)^*a*^	87.4 (43.7)	88.3 (69.3)
Redundancy	6.0	6.8
No. of reflections	92,015	780,730
*R*_work_^*b*^	0.339	0.274
No. of atoms^*c*^		
Protein	7,029	7,369
Water	0	75
Average B factors		
Protein	73	32
Water	NA^*d*^	30
RMSD		
Bond lengths (Å)	1.04	1.10
Bond angles (**°**)	0.004	0.004
Ramachandran^*e*^		
Favored (%)	94.37	94.47
Allowed (%)	5.40	5.19
Outliers (%)	0.23	0.11
Poor rotamers (%)^*e*^	1.59	0.74
Cβ deviations (%)^*e*^	0	0
Clash score^*e*^	11.57	10.47
Molprobity score^*e*^	2.11 (100th percentile)	1.92 (98th percentile)

a The values in parentheses are for the highest-resolution shell.

b If calculated, the *R*_free_ value would have been very close to the *R*_work_ value due to the 5- and 15-fold NCS (79). Thus, all measured reflections were used in the crystallographic refinement.

c The values are for the icosahedral asymmetric unit.

d NA, not applicable.

e According to the criterion of Molprobity (80).

### Structure of the VP3 P domain.

The SBPV virion represents the first atomic structure of a virus from the family Iflaviridae. Unlike in the previously structurally characterized viruses from the order Picornavirales, the SBPV capsid protein VP3 contains a C-terminal extension of residues 267 to 430 (15) that fold into the globular P domain positioned on the capsid surface (Fig. 1C and D). The domain consists of a central twisted antiparallel β-sheet formed from strands β4, β5, and β6 surrounded by the 14-residue-long α-helix α1, 3-residue-long 3.10 helix, and two shorter β-sheets containing strands β1 and β2 and β3 to β7 (Fig. 1D). The β-strands are connected by loops that vary in length between 6 and 23 residues. In both of the crystal forms, the residues of the P domain have higher average B factors (crystal 1 B = 110 Å^2^; crystal 2 B = 57 Å^2^) than the average B factors of the rest of the capsid (crystal 1 B = 57 Å^2^; crystal 2 B = 16 Å^2^), indicating a higher mobility of the P domain. The P domains in the two crystal forms are similar, with an RMSD of 0.32 Å for 144 Cα atoms.

**FIG 2:**
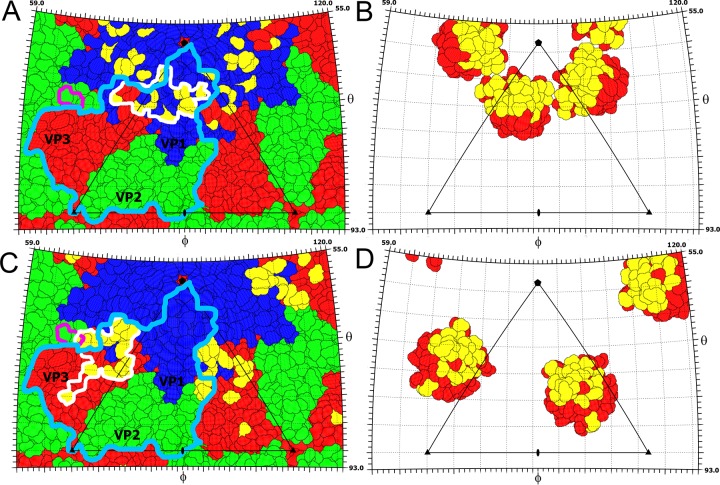
Interactions of P domain with the core of the SBPV capsid. (A and B) P domain footprints on the SBPV surface in crystal forms 1 (A) and 2 (B). The images show two-dimensional projections of the SBPV virion surface without the P domains. Residues of capsid proteins VP1, VP2, and VP3 are outlined in blue, green, and red, respectively. Residues involved in interaction with the P domain are shown in yellow. The P domain footprints are outlined by white lines. The border of one VP2-VP3-VP1 protomer is indicated by a light blue line. RGD residues of VP2 are indicated by a magenta line. (C and D) Inner surfaces of P domains in crystal forms 1 (C) and 2 (D), viewed from inside the particle. Residues interacting with the core of the capsid are shown in yellow and the remaining residues in red. Positions of 2-fold, 3-fold, and 5-fold icosahedral symmetry axes are shown as ovals, triangles, and pentagons, respectively. One icosahedral asymmetric unit is outlined by a triangle.

**FIG 3:**
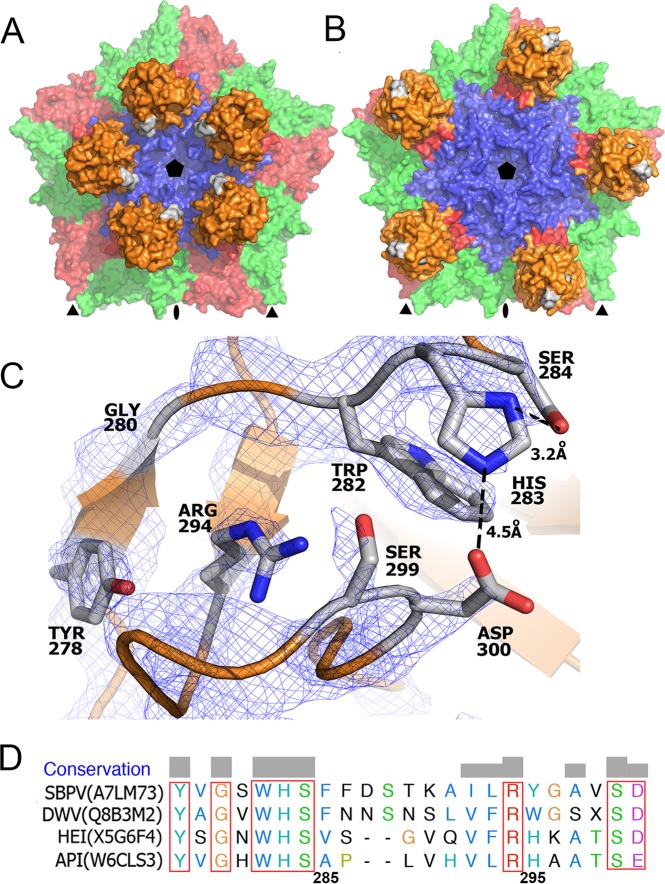
The P domain contains a putative Ser-His-Asp active site that is part of a patch of residues that are conserved among iflaviruses. The conserved residues are highlighted in gray in pentamers of capsid protein protomers in the conformation from crystal form 1 (A) and crystal form 2 (B). Detail of the putative active site with electron density contoured at 2σ (C). A sequence alignment of residues forming the conserved patch in the P domain is also shown (D). HEI, heliconius erato iflavirus; API, antherae pernyi iflavirus. Uniprot accession numbers of the sequences used in the alignment are provided.

The P domains are positioned in different locations on the virion surface in the two crystal forms (Fig. 1 and 2). It is important to note that the domains are not held in position by crystal contact in either of the crystal forms. In crystal form 1, five P domains related by one icosahedral 5-fold axis form a “crown” on the virion surface (Fig. 1A and 3A). The crowns have a diameter of 90 Å and protrude 50 Å above the capsid surface, giving the SBPV virion its characteristic shape (Fig. 1A). Residues from loop β2-β3 as well as the N- and C-terminal loops and β2 of the P domain interact with the BC, CD, and EF loops of VP1, forming an interface with a buried surface area of 850 Å^2^ (Fig. 2A and B). P domains within the same crown do not interact with each other (Fig. 1A and 3A). In crystal form 1, the electron density map corresponding to the P domains is less well ordered than that of the rest of the SBPV virion, indicating an increased mobility of the crown.

In crystal form 2, the P domain is positioned approximately equal distances from the icosahedral 5-fold, 3-fold, and 2-fold axes (Fig. 1B and 3B). Residues from α1, β3, β5, β7, and loops β2-β3, β3-β4, and β4-β5 of the P domain interact with the CD and GH loops of VP3, the C terminus of VP1, and the GH loop of VP2, forming an interface with a buried surface area of 1,150 Å^2^ (Fig. 2C and D). The density of the P domain is better resolved than in crystal form 1, indicating that the P domain forms more stable interactions with the capsid surface at the interface observed in crystal form 2. The transition between the two alternative positions of the P domain on the virion surface requires a 122° rotation of the domain around the axis, which passes through Lys266 (Fig. 1C). The center of mass of the P domain in crystal form 2 is shifted 36 Å toward the 3-fold axis relative to its position in crystal form 1 (Fig. 1). This movement of the domain is possible due to a 23-residue-long flexible linker that connects the P domain to the core of the VP3 subunit.

The crystallization conditions that produced the two crystal forms of SBPV differed in terms of solution components and pH, which was 6.5 for crystal form 1 and 4.5 for crystal form 2 (Table 1). We speculate that the differences in localization of the P domains might be induced by the differences in the crystallization conditions. Furthermore, it is possible that the two observed locations of the P domain on the virion surface reflect movements of the domain required for SBPV cell entry *in vivo*. Similar mobility of the protruding domain was previously reported for capsid proteins of mammalian caliciviruses, where it was speculated to facilitate virus-receptor interactions (38–40). The cell entry of iflaviruses has not been studied, but it is likely to involve receptor-mediated endocytosis as has been described for mammalian picornaviruses (41, 42). The endosomal entry involves exposure of the virions to low pH that could trigger movements of the P domain that might be required for cleavage of substrate by the putative catalytic triad within the P domain as described below.

### The P domain contains a putative receptor-binding or catalytic site.

Residues Ser284, His283, and Asp300 from the P domain of VP3 are located close to each other, indicating the presence of a putative catalytic triad (43) that might be involved in the cleavage of an as-yet-unknown substrate. These residues face the interior of the crown in crystal form 1; however, they constitute the apex of the P domain in crystal form 2 (Fig. 3A and B). The distances between the side chains of the putative reactive site are larger than ideal for catalyzing the hydrolytic reaction (Fig. 3C) (43). Nevertheless, it is possible that the optimal configuration of the active site might be achieved upon binding the unknown substrate to the P domain. This type of catalytic triad has been previously identified in proteases, lipases, and esterases (43–45). The residues constituting the putative active site are conserved among other iflaviruses that have P domains, including DWV, VDV-1, and Kakugo virus (20, 46, 47). However, the iflaviruses Sacbrood and Perina nuda virus lack P domains altogether (48, 49). Catalytic activity of the putative active site might be required for the virions to escape from endosomes in a manner analogous to the lipase activity present in the N-terminal domain of capsid proteins of parvoviruses (50). There are five additional conserved residues located in the vicinity of the putative active site in strand β1 and loops connecting strands β1-β2 and β2-β3 (Fig. 3C). This is in contrast to the overall 12% sequence identity of the P domains. The conservation of the residues reinforces the possibility that they may constitute a receptor or substrate-binding site. Furthermore, a similar conserved patch of residues in P domains of noroviruses was shown to bind glycans (51, 52). Additional experiments are required to identify the putative receptor of SBPV and to determine whether the catalytic triad cleaves it.

**FIG 4:**
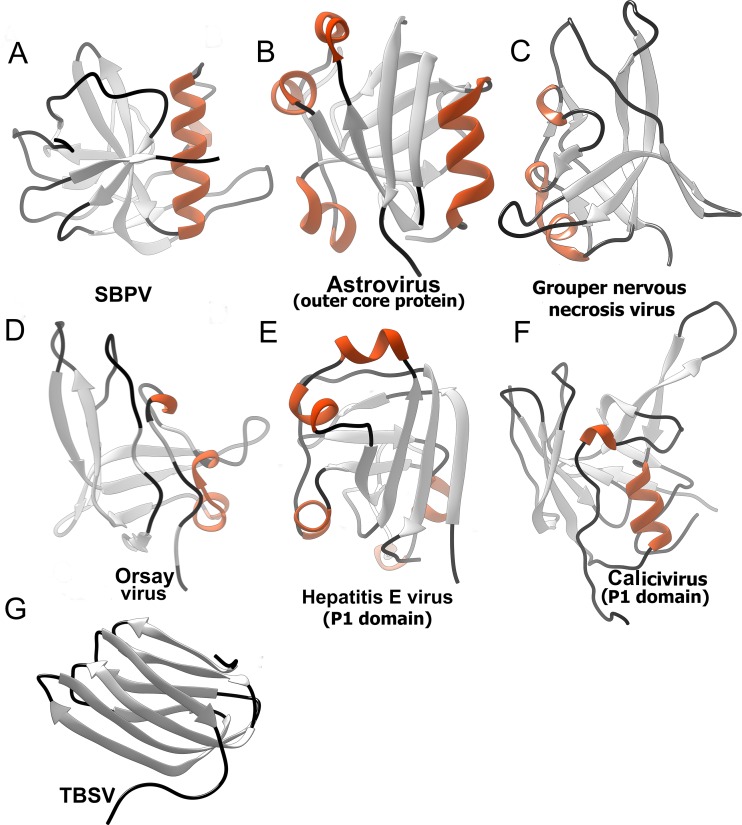
Protruding domains of viruses identified in a DALI search based on similarity to SBPV P domain. (A) SBPV; (B) human astrovirus outer coat protein (5EWN) (55); (C) grouper nervous necrosis virus (4RFU) (56); (D) orsay virus (4NWW) (57); (E) P1 domain of human hepatitis E virus (3HAG) (76); (F) P1 domain of human calcivirus (2GH8) (77). Protruding domain of tomato bushy stunt virus (2TBV) (78) is shown for comparison, however, it was not identified in the DALI server search. β-Strands are shown in light gray, helices in orange, and loops in black.

The DALI server was used to identify structures similar to the P domain (Table 2) (53). Most of the top hits were domains of virus capsid proteins that are exposed on the virion surface and therefore might be involved in receptor binding or cell entry. A common feature of these domains is a core formed of β-strands that is in some cases complemented by one or more short α-helices located at the periphery of the domain (Fig. 4). Furthermore, the P domains were also found in plant picorna-like viruses from the family Tombusviridae (54). In these species, however, the protrusions exhibit a β-jellyroll fold. Even though the surface domains could be identified in the DALI search, the structures of the domains are quite different and cannot be meaningfully superimposed. The surface domains were identified in viruses from the families Tombusviridae, Nodaviridae, Hepeviridae, and Astroviridae (54–57). All these viruses have positive-sense single-stranded RNA (ssRNA) genomes and similar overall virion architectures. It is therefore possible that a common ancestor of these viruses contained the P domain. However, the P domains were retained in the evolution of only some of the viruses.

**TABLE 2:** DALI search identification of proteins similar to the SBPV P domain

Structure	PDB code	DALI Z score	RMSD	Sequence identity (%)
Human astrovirus capsid protein	5ewn	4.5	3.6	9
P domain of grouper nervous necrosis virus	4rfu	4.2	3.6	5
Orsay virus	4nww	3.3	4.5	9
Hepatitis E virus capsid protein	2zzq	3.0	3.3	13

### Putative SBPV integrin receptor binding site.

Currently there is no information about the cell entry of honeybee viruses, and the putative receptors remain to be identified. However, the VP2 subunit of SBPV contains the integrin recognition motif Arg-Gly-Asp (RGD) in the GH loop (Fig. 1C). The GH loop is exposed on the virion surface in crystal form 1 but is partly covered by the P domain in crystal form 2 (Fig. 2A and B). Integrins serve as cell entry receptors for numerous viruses, including human picornaviruses such as the foot-and-mouth disease virus (FMDV) and several parechoviruses (58–60). The RGD motif within FMDV is located in the VP2 subunit, similar to the case with SBPV, although closer to the icosahedral 2-fold axis (Fig. 1C). The RGD motif is not conserved across different iflaviruses and may confer specific tissue tropism to SBPV. Even though honeybees encode a number of integrins (61), their involvement in virus cell entry has not been demonstrated so far.

### Decreased pH does not induce formation of SBPV A particles.

Picornaviruses enter cells through receptor-mediated endocytosis. The receptor binding and low pH of endosomes were shown to trigger the formation of expanded A particles and the subsequent genome release of many picornaviruses (62). The A particles are characterized by a 5 to 10% increase in virion radius and the formation of holes in the capsid (42, 63–66). However, the SBPV virion structures determined at pH 6.5 and 4.5 are nearly identical in size (Table 3). Therefore, it appears that the pH (4.5) of the crystallization condition was not sufficient to induce formation of the SBPV A particles. The induction of SBPV genome release might require binding to a receptor, or iflaviruses might use an entirely different mechanism for genome release.

**TABLE 3:** Comparison of size and volume of SBPV particles determined in crystal forms 1 and 2

Crystal form	Mean virion radius (Å)^*a*^	Virion vol (Å^3^)^*b*^
1	140	6.385 × 10^6^
2	139	6.386 × 10^6^

a Determined as distance of the center of mass of the icosahedral asymmetric unit from the particle center.

b Volume of virion cavity calculated based on virion structures. The space occupied by the unstructured parts of the capsid proteins located on the inside of the capsid was calculated based on average amino acid volumes and subtracted from the cavity volume.

### Comparison to virion structures of dicistroviruses.

**FIG 5:**
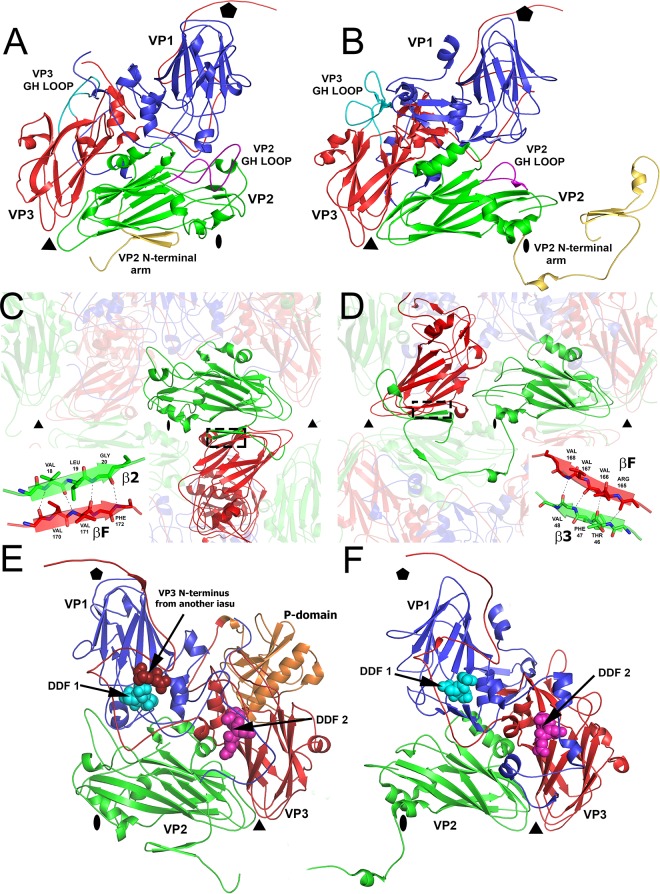
Comparison of SBPV structure to that of dicistrovirus TrV. (A and B) Cartoon representations of icosahedral asymmetric units of SBPV (A) and TrV (B). VP1 subunits are shown in blue, VP2 in green, and VP3 in red. The GH loop of VP2 is highlighted in magenta, the GH loop of VP3 is in cyan, and the N-terminal arms of VP2 are in yellow. (C and D) Domain swapping between SBPV (C) and TrV (D) N-terminal arms of VP2 subunits that mediate interpentamer interactions. The insets show details of hydrogen bonds between β2 of VP2 and βF of VP3. (E and F) Location of DDF sequences, which might be involved in the cleavage of VP0 to VP4 and VP3, on the inside of the capsid of SBPV (E) and TrV (F).

The most notable difference between SBPV and structurally characterized dicistroviruses, besides the P domain, is in the positioning of the N-terminal arm of the VP2 protein, which contributes to the interpentamer contacts within the capsid (Fig. 5A to D). In SBPV, two β-strands from the N-terminal arm of VP2 extend the β-sheet CHEF of a VP3 from the neighboring pentamer (Fig. 5C). In contrast, in dicistroviruses represented by TrV and cricket paralysis virus (CrPV), the N-terminal arm of the VP2 subunit reaches around an icosahedral 2-fold axis into the neighboring pentamer, approaches a 3-fold axis, and forms two β-strands that extend the β-sheet CHEF of a VP3 subunit from the same pentamer (Fig. 5D) (67, 68). Thus, the VP2 N-terminal arms of SBPV and dicistroviruses mediate interactions between VP2 and VP3 subunits in different relative positions within their virions. However, the type of interaction, i.e., extension of the β-sheet CHEF of VP3, is the same for both the viruses, representing domain swapping of the VP2 N-terminal arms. It was speculated previously that the observation of domain swapping among homologous complexes is indicative of hinge movements of structural units connected by the swapped domains. The alternative placements of the N-terminal arms of VP2 subunits therefore indicate that pentamers of capsid proteins could move relative to each other.

Additional differences between SBPV and dicistroviruses can be found on the capsid surface. The RGD containing the GH loop of the SBPV VP2 subunit contains 30 residues, while in TrV and CrPV it is only 17 residues long (Fig. 5A and B) (67, 68). The SBPV loop therefore elevates higher above the surface of the virion, which might be required for binding to the putative integrin receptor (Fig. 1C). On the other hand, the GH loop of the VP3 subunit is longer in TrV, containing 36 residues in comparison to 24 in SBPV (Fig. 5A and B) (68).

The maturation of capsids of viruses from the order Picornavirales is connected to a cleavage of capsid protein VP4 from the N terminus of a precursor subunit, called VP0. In picornaviruses, VP0 cleavage generates the proteins VP4 and VP2, while it was suggested that in iflaviruses the precursor cleavage produces VP4 and VP3 (67, 68). It has been proposed that a conserved Asp-Asp-Phe (DDF) motif, present in parts of capsid proteins that are exposed to the virion cavity, is involved in the VP0 cleavage (67–69). The dicistroviruses CrPV and TrV contain the DDF sequence in a loop immediately following β-strand I of VP1, while TrV has an additional DDF sequence, in a loop following β-strand I of VP3 (Fig. 5F) (67, 68). SBPV also has two DDF sequences. One is in VP1, residues 226 to 228, and the second one is formed by residues 239 to 241 of VP3 (Fig. 5E). Therefore, the locations of the DDF sequences in SBPV are similar to those in TrV (Fig. 5E and F). The DDF site in VP1 subunit of SBPV is located within 4 Å of the N terminus of VP3 subunit from a neighboring protomer, suggesting that it might mediate the VP0 maturation cleavage (Fig. 5E).

### Absence of a hydrophobic pocket in VP1.

The VP1 subunits of enteroviruses and several other vertebrate picornaviruses were indicated to contain a hydrophobic pocket that might bind a putative lipid-like molecule called the “pocket factor” (70, 71). Pocket factor mimetics that bind into the VP1 pocket with high affinity were shown to inhibit the infection of some picornaviruses (72–75). However, such a hydrophobic pocket is not formed within the VP1 subunits of SBPV. This suggests that capsid binding inhibitors may not be effective as antivirals against honeybee viruses. However, compounds targeting the putative His-Ser-Asp catalytic or receptor binding site in the P domain may prevent the infection of iflaviruses containing P domains.
